# Acidithiobacillus acidisediminis sp. nov., an acidophilic sulphur-oxidizing chemolithotroph isolated from acid mine drainage sediment

**DOI:** 10.1099/ijsem.0.005868

**Published:** 2024-05-28

**Authors:** Xiu-Tong Li, Zong-Lin Liang, Ye Huang, Zhen Jiang, Zhen-Ni Yang, Nan Zhou, Ying Liu, Shuang-Jiang Liu, Cheng-Ying Jiang

**Affiliations:** 1 State Key Laboratory of Microbial Resources, Institute of Microbiology, Chinese Academy of Sciences, Beijing 100101, PR China; 2 University of Chinese Academy of Sciences, Beijing 100049, PR China; 3 Innovation Academy for Green Manufacture, Chinese Academy of Sciences, Beijing 100190, PR China; 4 State Key Laboratory of Microbial Biotechnology, Shandong University, Tsingdao 266237, PR China

**Keywords:** *Acidithiobacillus*, *Acidithiobacillia*, sulphur-oxidizing, acid mine drainage, acidophile

## Abstract

Strain S30A2^T^, isolated from the acid mine drainage sediment of Mengzi Copper Mine, Yunnan, is proposed to represent a novel species of the sulphur-oxidizing genus *Acidithiobacillus*. Cells were Gram-stain-negative, non-endospore forming, highly motile with one or two monopolar flagella and rod-shaped. The strain was mesophilic, growing at 30–50 °C (optimum, 38 °C), acidophilic, growing at pH 2.0–4.5 (optimum, pH 2.5), and tolerant of 0–4 % (w/v; 684 mol l^−1^) NaCl. The 16S rRNA gene-based sequence analysis showed that strain S30A2^T^ belongs to the genus *Acidithiobacillus* and shows the largest similarity of 96.6 % to the type strain *Acidithiobacillus caldus* KU^T^. The genomic DNA G+C content of strain S30A2^T^ was 59.25 mol%. The average nucleotide identity ANIb and ANIm values between strain S30A2^T^ and *A. caldus* KU^T^ were 70.95 and 89.78 %, respectively and the digital DNA–DNA hybridization value was 24.9 %. Strain S30A2^T^ was strictly aerobic and could utilize elementary sulphur and tetrathionate to support chemolithotrophic growth. The major cellular fatty acid of S30A2^T^ was C_19 : 1_ω7*c*. The respiratory quinones were ubiquinone-8 and ubiquinone-7. Based upon its phylogenetic, genetic, phenotypic, physiologic and chemotaxonomic characteristics, strain S30A2^T^ is considered to represent a novel species of the genus *Acidithiobacillus*, for which the name *Acidithiobacillus acidisediminis* sp. nov. is proposed. The type strain is S30A2^T^ (=CGMCC 1.17059^T^=KCTC 72580^T^).

## Introduction

With the application of 16S rRNA gene sequence analysis and DNA–DNA hybridization technology, Kelly and Wood [1] reclassified the genus *Thiobacillus* in 2000 and created *Acidithiobacillus* in the *Acidithiobacillaceae* of the *Acidithiobacillales*, which was an order insertae sedis, until Williams and Kelly [2] determined that the order was neither in the class *Gammaproteobacteria* nor the *Betaproteobacteria* and proposed the class *Acidithiobacillia* in what is now the *Pseudomonadota* [1,2]. The genus includes 10 species [3]: *Acidithiobacillus thiooxidans* [4], *Acidithiobacillus concretivorus* [5], *Acidithiobacillus ferrooxidans* [6], *Acidithiobacillus albertensis* [7], *Acidithiobacillus caldus* [8], *Acidithiobacillus ferrivorans* [9], *Acidithiobacillus ferridurans* [10], *Acidithiobacillus ferriphilus* [11], *Acidithiobacillus sulfuriphilus* [12] and *Acidithiobacillus ferrianus* [13]. *A. thiooxidans* is the type species proposed by Waksman and Joffe [4]. All *Acidithiobacillus* species isolated thus far are obligate acidiophiles with pH optima ranging from pH 2.0 to 4.0 [2]. The genus comprises Gram-stain-negative small rods (0.3–0.9×0.7–2.5 µm) that do not produce endospores, exospores or cysts. Most are motile by one or more flagella. The genus comprises two key functional guilds: obligate aerobes that grow on thiosulphate, polythionates and/or elementary sulphur (*A. thiooxidans*, *A. albertensis*, *A. concretivorus*, *A. caldus* and *A. sulfuriphilus*) and facultative anaerobes that grow on tetrathionate, elementary sulphur and/or ferrous iron (or minerals containing it) and can respire ferric iron, but only when growing on sulphur species (*A. ferrooxidans*, *A. ferridurans*, *A. ferrivorans*, *A. ferriphilus* and *A. ferrianus*) [14]. The genus consists of moderate thermophiles represented by *A. caldus* KU^T^ (optimum growth at 45 °C) and mesophiles represented by *A. thiooxidans* ATCC 19377^T^ (optimum temperature 25–35 °C) [12]. All the species in *Acidithiobacillus* contain ubiquinone-8 (UQ-8) as their main respiratory quinones [2]. The genus *Acidithiobacillus* is widespread in acid mine environments including acid mine drainage, waste ores, sediments, and biofilms surrounding the mines [10]. Previous studies showed that the reason why *Acidithiobacillus* species survive in these extreme environments is due to their strong ability of metal resistance [15]. Futhermore, all the species can oxidize elementary sulphur and/or sulphur oxyanions (thiosulphate, polythionates) autotrophically in extreme environments [2].

Here we describe a novel member of the genus *Acidithiobacillus*, strain S30A2^T^, which was isolated from sediment of acid mine drainage environment from Mengzi Copper Mine, Yunnan, PR China. We present phylogenic, genomic, physiologic and chemotaxonomic data to support its recognition as a novel species of *Acidithiobacillus*.

## Isolation and ecology

Strain S30A2^T^ was isolated from an acid sediment sample of Mengzi Copper Mine (103° 46′ 47″ N 23° 28′ 53″ E; at an altitude of 1.847 km), Yunnan Province, PR China. The sample was collected on 22 September 017 at 25 °C and pH 2.37±0.05.

The sample was first incubated in enrichment medium at 30 °C for 1 week. The enriched culture was diluted serially to 10^−4^, 10^−5^ and 10^−6^ fold of the original culture, then spread on the solid isolation medium plates and incubated at 30 °C for obtaining individual colonies. The culture media including enrichment medium and isolation medium were established by adjusting the components and pH of the medium based on Johnson’s previous studies [16,17]. The basal salt solution of the enrichment medium (BSS1) contained (g l^–1^): (NH_4_)_2_SO_4_ (0.45), MgSO_4_·7H_2_O (0.50), Na_2_SO_4_·10H_2_O (0.15), KH_2_PO_4_ (0.05 g l^–1^), KCl (0.05), Ca(NO_3_)_2_·4H_2_O (0.014) and 1 ml trace elements solution; 3 mol l^–1^ H_2_SO_4_ solution was used to adjust the pH to 2.5. The trace elements solution contained (mg l^–1^) ZnSO_4_·7H_2_O (10), CuSO_4_·5H_2_O (1000), MnSO_4_·4H_2_O (1000), CoSO_4_·7H_2_O (1000), Cr_2_(SO_4_)_3_·15H_2_O (500), Na_2_B_4_O_7_·10H_2_O (500), Na_2_MoO_4_·2H_2_O (500) and NaVO_3_ (100) and then stored in a dark place. BSS1 was autoclaved at 121 °C for 20 min and was supplemented with 0.05 filter-sterilized FeSO_4_ (pH 1.5), 0.02 % (w/v) filter-sterilized yeast extract and 1.0 % (w/v) tyndalized precipitated sulphur. The basal salt solution of the isolation medium (BSS2) contained (g l^–1^): (NH_4_)_2_SO_4_ (3.00), MgSO_4_·7H_2_O (0.50), Na_2_SO_4_·10H_2_O (0.15), KH_2_PO_4_ (0.10), KCl (0.10), Ca(NO_3_)_2_·4H_2_O (0.014) and 1 ml trace elements solution, adjusted the pH to 2.7. The liquid isolation medium contained BSS2, 0.05 mmol l^−1^ FeSO_4_, 0.02 % (w/v) yeast extract and 3.0 % (w/v) precipitated sulphur (Sinopharm Chemical Reagent Co., Ltd., No. 20210526), pH 3.5. To prepare the solid isolation medium, a two-fold BSS2 and 12.0 g l^–1^ gellan gum (Gelrite, Sigma-Aldrich) solution was autoclaved at 121 °C for 20 min separately. Sterilized BSS2 was supplemented with filter-sterilized 0.05 mmol l^−1^ FeSO_4_, 0.02 % (w/v) yeast extract and 5 mmol l^−1^ K_2_S_4_O_6_ cooled down to about 60 °C and mixed with equal volume 12.0 g l^–1^ gellan gum solution. *A. caldus* KU^T^ (=DSM 8584^T^) was obtained from the German Collection of Microorganisms and Cell Cultures GmbH (DSMZ) as a reference strain for the contrast experiments. The broth of *A. caldus* KU^T^ (Medium 150 a) contained (g l^–1^): (NH_4_)_2_SO_4_ (3.00), MgSO_4_·7H_2_O (0.50), K_2_HPO_4_·3H_2_O (0.50), KCl (0.10), Ca(NO_3_)_2_ (0.01) precipitated sulphur (5.00) and 10.00 ml trace element solution which contained (mg l^–1^) FeCl_3_·6H_2_O (1100), CuSO_4_·5H_2_O (50), H_3_BO_3_ (200), MnSO_4_·H_2_O (200), Na_2_MoO_4_·2H_2_O (80), CoCl_2_·6H_2_O (60) and ZnSO_4_·7H_2_O (90). All the physiologic and chemotaxonomic tests of S30A2^T^ were carried out together with *A. caldus* KU^T^ at the same culture conditions in Medium 150 a.

## Phylogeny

Approximately 500 ml culture incubated in the liquid Medium 150 a at 30 °C for 5 days was filtered using glass microfiber filters (Whatman, Art. No. 1083–047) to remove sulphur powder and then centrifuged at 10 000 g to collect cells. The genomic DNA of strain S30A2^T^ was extracted with the Wizard Genomic DNA Purification Kit (Promega) and sequenced with Illumina NovaSeq 6000 and PacBio Sequel platforms at Guangdong Magigene Biotechnology Co., Ltd. (www.magigene.com) following the standard methods. The maximum-likelihood tree on the basis of full-length 16S rRNA (rrs) gene sequences from genomes of the type strains within the *Acidithiobacillales* from the GenBank database (www.ncbi.nlm.nih.gov/genbank/) was reconstructed in mega 7 with partial deletion of gaps (95 % cut-off). Sequences were aligned using muscle [18]. Model testing was based on the lowest corrected Akaike information criterion (AICc). The Tamura–Nei model was used with a gamma distribution (five discreet gamma categories) with invariant sites (G+I) [19,21]. Genome sequences of the type strains within *Acidithiobacillales* were downloaded from the NCBI database (www.ncbi.nlm.nih.gov/genome/). The phylogenetic tree based on genomes was reconstructed using amino acid sequences of 92 concatamer proteins among strains S30A2^T^ and other related type strains within *Acidithiobacillales* using UBCG pipeline [22].

**Fig. 1.**
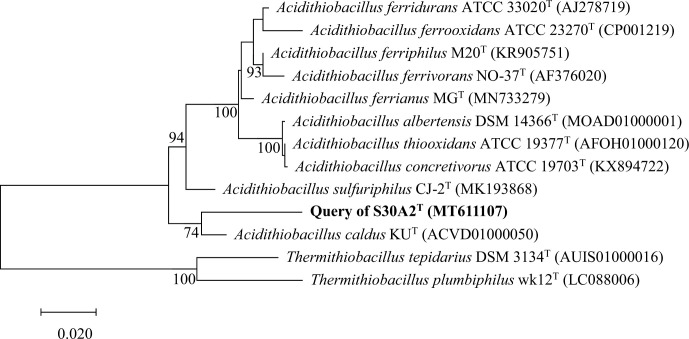
Maximum-likelihood phylogenetic tree of strain S30A2^T^ based on the full-length 16S rRNA gene sequences. The genus *Thermithiobacillus* is used as an outgroup. The results show the positions of strain S30A2^T^ (highlighted in bold) and all type strains within *Acidithiobacillales*. Bootstrap values (300 replicates) are shown next to the branches, values ≥70 % were shown. GenBank accession numbers are given in parentheses. The scale bar 0.020 represents the average number of nucleotide substitutions per site.

Strain S30A2^T^ was phylogenetically related to members of the genus *Acidithiobacillus* with identity in the range of 94.7–96.6 %, which was below the classification threshold of 98.7 % for potential novel species and above the classification threshold of 94.5 % for a potential novel genus, and the closest relative was the type strain *A. caldus* KU^T^ [23]. As the phylogenetic trees show in Figs1  2, the topological structures of the maximum-likelihood and ribosomal concatamer proteins trees are similar and strain S30A2^T^ clusters together with *A. caldus* KU^T^ into a branch indicating that strain S30A2^T^ represents a novel species belonging to the genus *Acidithiobacillus*.

**Fig. 2.**
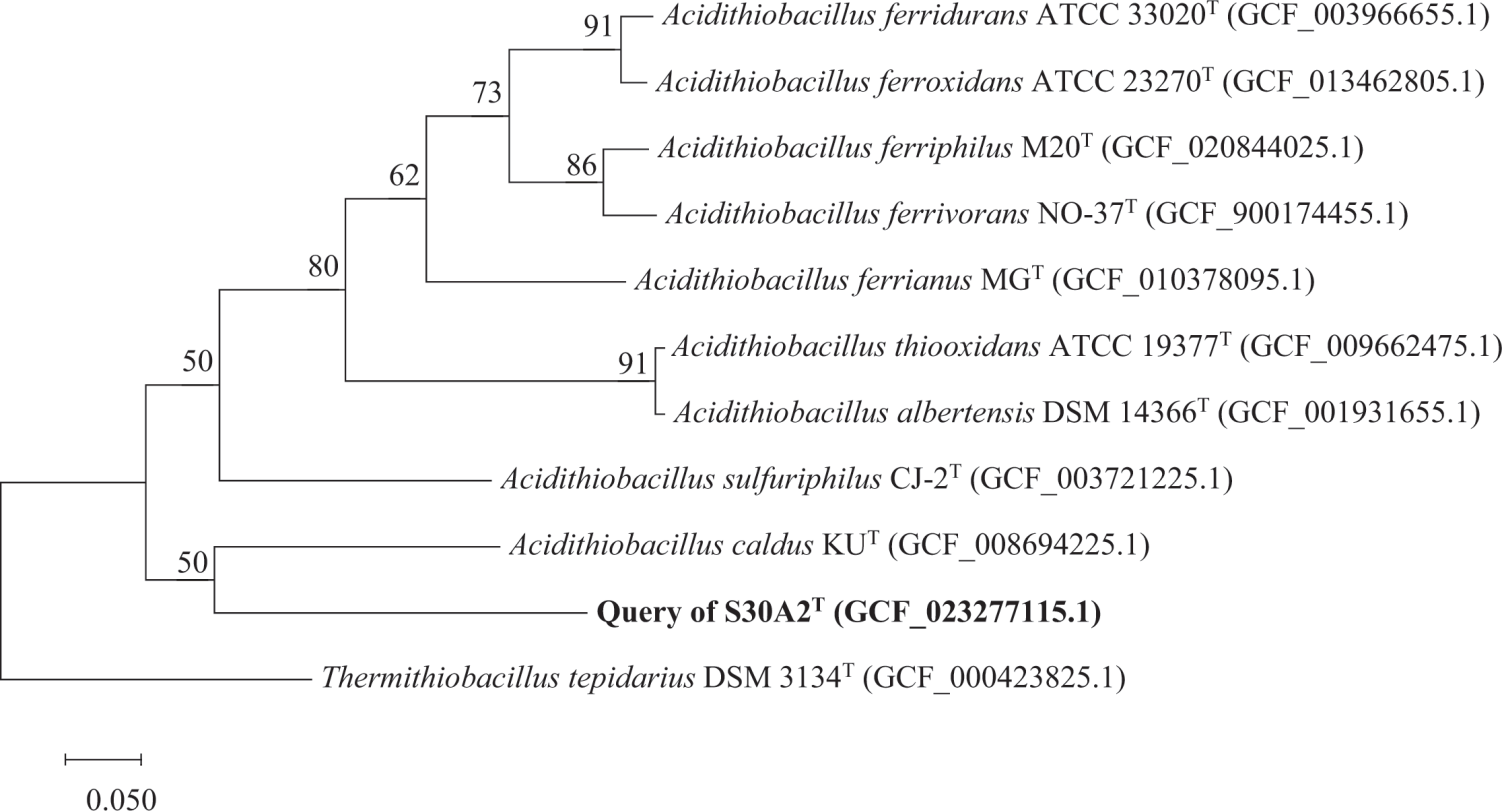
The phylogenetic tree based on genomes was reconstructed using the amino acid sequences of 92 concatamer proteins of strains S30A2^T^ (highlighted in bold) and other related type strains within *Acidithiobacillales. Thermithiobacillus tepidarius* DSM 3134^T^ (GCF_000423825.1) is used as an outgroup. Genome sequence accession numbers are given in parentheses. Numbers at branch nodes represent confidence levels, values ≥50 % were shown. Bar, 0.050, represents the number of substitutions per site.

## Genome features

Illumina and PacBio Sequel sequencing platforms were used for DNA sequencing at Guangdong Magigene Biotechnology Co., Ltd. (www.magigene.com). Data after quality control were filtered, assembled and optimized using SMRT Link version 5.1.0 (www.pacb.com/support/software-downloads/), Unicycle (https://github.com/rrwick/Unicycler) and Arrow software to obtain the complete genome sequences [24]. Coding gene prediction was performed using Glimmer3 [25]. Non-coding RNA (ncRNA) contained sRNA, rRNA and tRNA was predicted by tRNAscan-SE 1.3.1 (http://lowelab.ucsc.edu/tRNAscan-SE/), rRNAmmer 1.2 (http://www.cbs.dtu.dk/services/RNAmmer/) and Cmsearch software, respectively. Analyses of prophage, genomics islands (GIs) and CRISPR (Clustered Regularly Interspaced Short Palindromic Repeat Sequences) were proceeded by phast, IslandPath-DIOMB and CRISPRdigger. The average of the reciprocal average nucleotide identity using blast (ANIb) and average nucleotide identity using MUMmer (ANIm) values were calculated via the online website JSpeciesWS (http://jspecies.ribohost.com/) [26]. Digital DNA–DNA hybridization (dDDH) calculations using whole genome sequences were conducted using DSMZ’s online service (http://ggdc.dsmz.de/) [27,28]. Functional gene annotation was provided by the COG (cluster of orthologous group of proteins) (www.ncbi.nlm.nih.gov/COG/) and KEGG (Kyoto Encyclopedia of Genes and Genomes) databases (www.genome.jp/kegg/kegg2.html) [29].

The final assembled genome of strain S30A2^T^ is composed of a circular chromosome with a length of 2.78 Mb and three circular plasmids of 1.52, 1.19 and 0.44 kb, respectively. The G+C contents of the chromosome and plasmids DNA were 58 mol%, 50 mol%, 58 mol%, and 60 mol%, respectively. Overall 2901 coding genes accounted for 91.13 % of the whole genome with total length of 2.56 Mb and average length of 884.06 bp. The genome contains 47 tRNA genes, two 5s rRNA genes, two 16s rRNA genes, two 23s rRNA genes and two sRNA genes. There are 20 GIs, 11 CRISPR and no prophage in the genome. The ANIb (70.95 %) and ANIm (89.78 %) values between strain S30A2^T^ and *A. caldus* KU^T^ are less than the interspecies threshold of 95 %, the dDDH value (24.9 %) is much lower than the interspecies threshold of 70 %, which indicated that S30A2^T^ represents a novel species of the genus *Acidithiobacillus*. Functional gene annotation results by the COG database showed that the main pathways detected in the genome of S30A2^T^ were related to cell wall/membrane/envelope biogenesis (with 213 genes), translation, ribosomal structure and biogenesis (186), general function prediction only (179), energy production and conversion (177), amino acid transport and metabolism (156), signal transduction mechanisms (151), inorganic ion transport and metabolism (147), replication, recombination and repair (146), function unknown (135) and so on (1088), detailed sequencing information is shown in Table S1, available in the online version of this article. The pathway analysed by KEGG showed that genes *fliDEFGHIKLMNOPQR* (K02407–K02412, K02414–K02421), *flgBCDEFGHIJKL* (K02387–K02397) and *flhAB* (K02400 and K02401) encoding a complete flagellar assembly pathway and genes *pilABCDFMNOPQRSTUVWY1Z* (K02650, K02652–K02654, K02656, K02662–K02672, K02674, K02676) encoding pilus assembly proteins were present in the genome of strain S30A2^T^. Genome annotation showed that the strain S30A2^T^ has an incomplete citrate cycle (TCA cycle) which lacks 2-oxoglutarate dehydrogenase E1 component (*sucA*, EC 1.2.4.2), 2-oxoglutarate dehydrogenase E2 component (*sucB*, EC 2.3.1.61) and succinate dehydrogenase (ubiquinone) flavoprotein subunit (*SDH1*, EC 1.3.5.1), indicating an obligately chemoautotrophic life for S30A2^T^, the incomplete TCA cycle was also detected in *A. caldus* KU^T^, in which genes for the 2-oxogluatarate dehydrogenase enzyme complex were absent. The complete Calvin–Benson cycle including the key enzyme ribulose-bisphosphate carboxylase (composed by Rubisco form I gene *CbbLS* and form II gene *CbbM*, EC 4.1.1.39) is found in the genome of S30A2^T^ and same predicted in KU^T^, suggesting the ability to fix carbon dioxide for energy acquisition [30]. Both S30A2^T^ and KU^T^ possess *soxAX*, *soxB*, *soxYZ* (K17222–K17223, K17224, K17226-K17227, encoding SOX system), *sqr* (K17218, encoding sulphide:quinone oxidoreductase, EC 1.8.5.4) and *sat* (K00958, encoding sulphate adenylyltransferase, EC 2.7.7.4), which were supposed to associate with sulphur metabolism. However, neither S30A2^T^ nor KU^T^ obtain the key enzymes sulphite oxidase (*SUOX*, EC 1.8.3.1) or adenosine phosphosulphate (APS) reductase (*aprAB*, EC 1.8.99.2) for oxidizing sulphite [31]. Different from KU^T^, S30A2^T^ lacks the genes for sulphur oxygenase/reductase (*sor*, EC 1.13.11.55), tetrathionate hydrolase (*tetH*, EC 3.12.1.B1) and thiosulphate:quinone oxidoreductase (*doxDA*, EC 1.8.5.2) [32]. Furthermore, S30A2^T^ lacks a putative membrane-associated nitrate reductase (*NarGHI*, EC 1.7.5.1) that is encoded by * A. caldus* KU^T^ for nitrogen assimilatory pathway.

## Physiology and chemotaxonomy

Cultures in 25 mmol l^−1^ K_2_S_4_O_6_ solution or 0.2 g l^–1^ precipitated sulphur were collected at the exponential stage to observe the cell morphology and the presence of pilus and flagellum using the method of phosphotungstic acid negative staining by transmission electron microscopy (JEOL, JEM-1400). Gram staining was carried by HYCX Gram staining kit (Art. No. G1060-100) following the instructions. The motility of strains was observed under a phase contrast microscope (Axio Imager A2, Zeiss). The presence of endospores was checked by the endospore stain kit (Solarbio, Art. No. G1132-2) following the instructions and judged by the colour change under an optical microscope. Cultures at the exponential stage were inoculated and detected after cultivation for 1 week to investigate the temperature range, pH range and salinity tolerance for growth. The temperature was set at 10, 15, 20, 25, 30, 35, 38, 40, 42, 45, 50, 55 and 60 °C respectively. The liquid media were adjusted to pH 0.5–6.0 at the gradient of 0.5 with H_2_SO_4_ or NaOH solution to test the pH growth range. The salinity tolerance of the strain was performed in the liquid medium with 0 %, 1 % (w/v; 171 mmol l^−1^), 2 % (w/v; 342 mmol l^−1^), 3 % (w/v; 513 mmol l^−1^), 4 % (w/v; 684 mmol l^−1^), 5 % (w/v; 855 mmol l^−1^) NaCl. Cultures at the exponential stage were inoculated in 150 a liquid medium added with Fe(II) (10 mmol l^−1^ FeSO_4_∙7H_2_O), precipitated sulphur (5 g l^–1^), 10 mmol l^−1^ Na_2_SO_3_, 10 mmol l^−1^ NaS_2_O_3_ and 5 mmol l^−1^ K_2_S_4_O_6_ as potential electron donors, the concentrations of Fe(II), Fe(III), SO_4_^2-^ and change of pH were measured after 1 week even 1 month to evaluate the oxidization capacities of iron and sulphur oxyanions (thiosulphate, polythionates) or elementary sulphur at oxic conditions. Fe(III) (10 mmol l^−1^ FeCl_3_∙6H_2_O), precipitated sulphur (5 g l^–1^) or SO_4_^2-^ (10 mmol l^−1^ Na_2_SO_4_) were separately supplemented as electron acceptors in liquid medium with 0.8 g l^–1^ yeast extract as electron donor and examined the colour change of lead acetate test paper and concentration of Fe(II) or SO_4_^2-^ at anoxic conditions to judge the reduction ability of the strains. The production of H_2_S was able to blacken lead acetate and the product was assayed by the lead acetate test paper (Art. No. RZK01472). The pH value was measured by a pH meter (Mettler Toledo). The concentration of Fe(II)/Fe(III) was detected using 1,10-phenanthroline spectrophotometry assay and the concentration of SO_4_^2-^ was determined using barium sulphate turbidimetric method by a portable colorimeter (DR890, Hach) following the instructions of the manufacturer [33,34]. The catalase activity was detected by dropping 5 % H_2_O_2_ solution on the bacterial lawn and observing whether bubbles were generated. If bubbles were generated immediately, the catalase activity was positive, otherwise negative. OX oxidase reagent (Art. No. 55635) was added to the colonies collected from solid plate and observed the colour change within 10–30 s corresponding oxidase reagent and ID colour catalase (bioMérieux). Cells at the logarithmic stage were coated on the plate, and the filter-paper discs treated with different antibiotics were applied on the coated plate. After 1 week of culture, the presence of inhibition rings indicated the bacteria were sensitive to the corresponding antibiotics, otherwise insensitive. Antibiotics of the filter-paper discs used in this study including (μg) amoxicillin (10), μg ampicillin (10), azithromycin (15), azlocillin (75), carbenicillin (100), cefaclor (30), cefazolin (30), cefepime (30), cefetamet (30), cefixime (5), cefmetazole (30), cefodizime (30), cefoperazone (75), cefoperazone (30), cefotaxime (30), cefpiramide (75), ceftazideme (30), ceftizoxime (30), cefuroxime (30), cephalothin (30), chloramphenicol (30), clindamycin (2), gentamicin (10), kanamycin (30), mezlocillin (75), neomycin (30), oxacillin (5), piperacillin (100), rifampin (5), spectinomycin (100), vancomycin (30), netilmicin (30), aztreonam (30) and 300 IU polymyxin B.

**Fig. 3.**
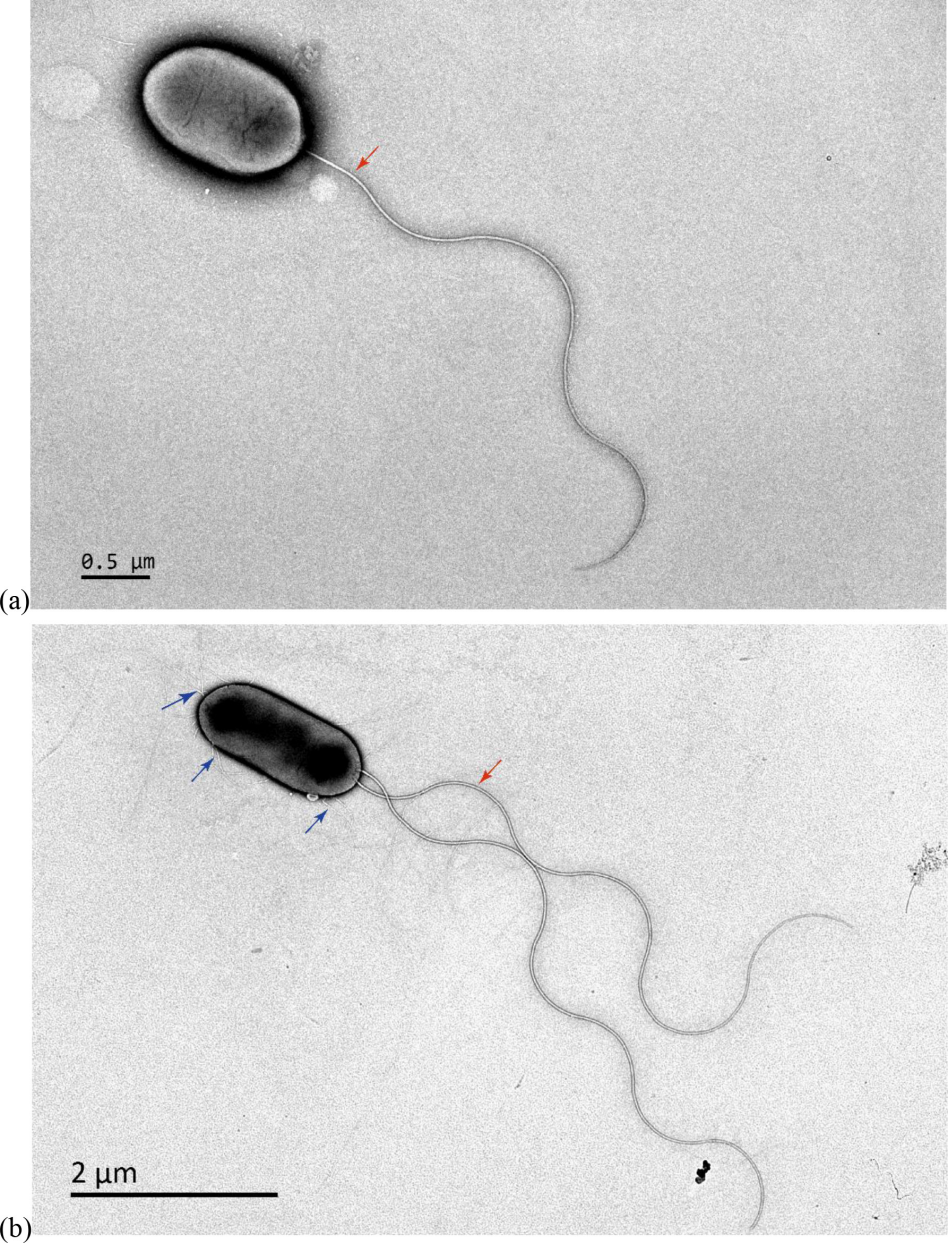
The transmission electron microscopy images of strain S30A2^T^ after cultured for 7 days in 25 mmol l^−1^ K_2_S_4_O_6_ (**a**) and 0.2 g l^−1^ precipitated sulphur (**b**). Red arrows indicate flagella and blue arrows indicate pili.

The colonies of strain S30A2^T^ are milky white, opaque, smooth, circular swollen and easy to pick up after 7 days cultured. S30A2^T^ is Gram-stain-negative. Cells of strain S30A2^T^ are straight rods with the size of 0.3–0.9×0.7–1.3 µm, highly motile with one or two polar flagella and cells differ in length and the amount of pili in K_2_S_4_O_6_ and precipitated sulphur (Fig. 3). No endospores is observed during the process of growth. To deeper understand the physiology and chemotaxonomy characteristics of strain S30A2^T^, physiological and biochemical results are compared with the type strains of other *Acidithiobacillus* species and detailed information are shown in Table 1. As with other members of the genus *Acidithiobacillus*, strain S30A2^T^ is acidophilic with a pH growth range of 2–4.5 and optimum pH of 2.5, can utilize elementary sulphur and tetrathionate as electron donors, and tolerant to high salty concentrations (highest at 4 %). Nevertheless, the growth temperature range (30–50 °C), optimum growth temperature (38 °C) of the strain are different from other species of the genus [2]. No growth is observed in the culture containing sodium thiosulphate or sodium sulphite. The strain failed to oxidize ferrous or pyrite and did not grow using ferric ion, sulphur or sulphate as electron acceptors. Cells of *A. caldus* KU^T^ were sensitive only to amoxicillin, azlocillin, carbenicillin, cefaclor, mezlocillin and piperacillin. However, cells of S30A2^T^ were sensitive to most antibiotics like amoxicillin, ampicillin, azlocillin, carbenicillin, cefaclor, cefepime, cefetamet, cefixime, cefodizime, cefoperazone, cefotaxime, cefpiramide, ceftizoxime, cefuroxime, cephalothin, mezlocillin, piperacillin, rifampin, spectinomycin and netilmicin (Table S2).

**Table 1.** Differential characteristics of strain S30A2^T^ and the reference type strains within *Acidithiobacillales* Strains: 1, S30A2^T^; 2, *Acidithiobacillus caldus* KU^T^ [8]; 3, *Acidithiobacillus albertensis* ATCC 35403^T^ [7,39]; 4, *Acidithiobacillus ferrianus* MG^T^ [13]; 5, *Acidithiobacillus ferridurans* ATCC 33020^T^ [10]; 6, *Acidithiobacillus ferriphilus* M20^T^ [11]; 7, *Acidithiobacillus ferrivorans* NO-37^T^ [9]; 8, *Acidithiobacillus ferrooxidans* ATCC 23270^T^ [6,9, 10, 40]; 9, *A. sulfuriphilus* CJ-2^T^ [12]; 10, *A. thiooxidans* ATCC 19377^T^ [4,9, 41]; 11, *A. concretivorus* ATCC 19703^T^ [5,42]; 12, *Thermithiobacillus tepidarius* DSM 3134^T^ [43]; 13, *Thermithiobacillus plumbiphilus* wk12^T^ [44]. +, Positive; –, negative; nd, not determined.

Characteristic	1*	2*	3	4	5	6	7	8	9	10	11	12	13
Cell morphology:													
Shape	Straight rods	Rods	Rods	Straight rods	Rods	Straight rods	Rods	Rods	Straight rods	Short rods	Straight rods	Short rods	Short rods
Size (μm)	0.3–0.9×0.7–1.3	0.8×1.8	0.45×1.2–1.5	1.2–2.5 (length)	0.3×1.2	1.0–2.0 (length)	0.5×2.4	0.5×1.0	0.5×1.5–2.5	0.5×1.0–2.0	0.5×1.0–2.0	0.2–0.4×0.6–1.0	0.5–1.0×0.7–2.2
Flagella	+	+	+	+	nd	nd	nd	+	+	nd	+	+	nd
Pilus	+	+	–	+	nd	nd	nd	+	+	–	nd	nd	nd
Motility	+	+	+	+	–	+	+	+	+	+	+	+	+
Gram stain	–	–	–	–	–	–	–	–	–	–	–	–	–
Growth conditions:													
Optimum pH	2.5	2.0–2.5	3.5–4.0	2.0	2.1	2.0	2.5	2.5	3.0	2.0–3.0	2.0–2.5	6.0–7.5	6.4–7.1
Optimum temperature (°C)	38	45	25–30	30	29	30	28–33	30–35	25–28	28–30	28	43–45	28–32
NaCl tolerance (%, w/v)	4 (684 mmol l^−1^)	4 (684 mmol l^−1^)	nd	3 (513 mmol l^−1^)	5 (855 mmol l^−1^)	3 (513 mmol l^−1^)	0.585 (100 mmol l^−1^)	3 (513 mmol l^−1^)	3 (513 mmol l^−1^)	5 (855 mmol l^−1^)	nd	0	0.702 (120 mmol l^−1^)
Growth substrates:													
Ferrous iron	–	–	–	+	+	+	+	+	–	–	–	–	nd
Thiosulphate	–	+	+	nd	+	+	+	+	+	+	+	+	+
Pyrite	–	–	–	+	+	+	+	+	–	–	nd	–	nd
Ferric iron	–	–	–	+	+	+	+	+	–	–	–	nd	nd
Chemotaxonomy compounds:													
Fatty acids (%)†	C_16 : 0_ (13.0), summed feature 8 (42.9)	Summed feature 8 (43.5), cyclo-C_19 : 0_ω8*c* (11.4), C_18 : 1_2-OH (10.6)	nd	C_16 : 0_ (17.6), C_18 : 1_ ω7*c* (24.1),summed feature 3 (23.1), summed feature 8 (24.1)	cyclo-C_19 : 0_ω8*c*, C_18 : 1_ ω7*c*, C_16 : 0_, C_16 : 1_, cyclo-C_17 : 0_, C_12 : 0_	C_18 : 1_ ω7*c*, C_16 : 1_, C_18 : 1_2-OH, C_14 : 0_3-OH, C_16 : 0_, C_12 : 0_	nd	C_18 : 1_ ω7c, C_16 : 1_, C_16 : 0_, cyclo-C_19 : 0_ω8*c*, C_14 : 0_, C_12 : 0_	C_18 : 1_ ω7*c* (33.8), C_18 : 1_2-OH (10.3), summed feature 2 (10.1), summed feature 3 (21.6)	Cyclo-C_19 : 0_, cyclo-C_17 : 0_, C_16 : 1_, C_18 : 1_, C_16 : 0_, C_14 : 0_3-OH	Cyclo-C_19 : 0_ω8*c*, cyclo-C_17 : 0_, C_16 : 0_, C_12 : 0_, C_14 : 0_3-OH, C_16 : 0_	C_16 : 0_, C_16 : 1_, cyclo-C_17 : 0_	C_16 : 1_, C_16 : 0_, C_18 : 1_
Respiratory quinones (%)	UQ-8 (67.7), UQ-7 (32.3)	UQ-8 (93.5), UQ-9 (0.3), UQ-7 (6.2)	UQ-8	UQ-8 (95), UQ-7 (5)	UQ-8	UQ-8 (94), UQ-9 (3),UQ-7 (2)	UQ-8	UQ-8	UQ-8 (98), UQ-7 (2)	UQ-8	UQ-8	UQ-8	UQ-8
Polar lipids‡	APL, PL, AL	DPG, PG, PE, AL, GL	nd	PG, PE, AL	PG, PE, AL	AL, PL, PG	nd	nd	AL, PG, PME	nd	nd	DPG, PE, PG, AGL	DPG, PE, PG, AGL
Genome features (compared to S30A2^T^):													
16S rRNA gene identity (%)*	100	96.61	95.18	95.11	95.11	94.85	94.80	94.73	95.71	95.26	95.50	91.42	91.04
G+C content (mol%)	59.25	63.9	61.5	58.2	58.0	57.4	55.0	58.0–59.0	61.5	52.0	nd	66.8	58.5
dDDH (%)§	100	24.90	19.40	20.30	22.20	22.00	25.80	22.40	21.60	20.80	nd	21.60	nd
ANIb (%)§	100	70.95	68.67	69.35	69.54	69.57	70.24	69.80	69.61	68.84	nd	67.60	nd

*Data from the present study.

†Summed feature 2 contains C_14 : 0_3-OH and/or iso-C_16 : 1_; summed feature 3 contains C_16 : 1_ ω7*c*, C_16 : 1_ ω6*c* and/or iso-C_15 : 0_2-OH; summed feature 8 contains C_18 : 1_ ω7*c* and/or C_18 : 1_ ω6*c*.

‡APL, unidentified aminophospholipid; PL, unidentified phospholipid; AL, unidentified aminolipid; DPG, diphosphatidylglycerol; PG, phosphatidylglycerol; PE, phosphatidylethanolamine; GL, unidentified glycolipid; PME, phosphatidylmethylethanolamine; AGL, aminoglycolipid.

§dDDH, digital DNA–DNA hybridization. ANIb, average nucleotide identity using blast.

Cells cultured in 150 a liquid medium were centrifuged and collected in a 50 ml centrifuge tube, frozen overnight at −80 °C and placed in a vacuum dryer until the cells were completely dehydrated. The fatty acids were methylated and analysed using gas chromatography (HP 6890 Series GC System, Hewlett Packard) [35]. Total lipids were extracted and separated by two-dimensional TLC plates (20×20 cm silica gel; Merck) with chloroform–methanol–water (65 : 24 : 4, v/v/v) in the first dimension, followed by chloroform–methanol–acetic acid–water (80 : 12 : 15 : 4, v/v/v/v) in the second dimension. The plates were sprayed with reagents to reveal corresponding lipids, including phosphomolybdic acid (5 % (w/v), dissolved in ethanol) for total lipids, molybdenum blue (Sigma) for phospholipids, ninhydrin (Sigma) for aminolipids and α-naphthol for glycolipids [36,37]. Respiratory quinones were extracted from freeze-dried cells with chloroform–methanol (2 : 1, v/v), purified by TLC and identified by HPLC equipped with a Zobax ODS C18 column (4.6×150 mm; Agilent) [38].

The polar lipids of S30A2^T^ contain two unidentified phospholipids, three unidentified aminophospholipids and an unidentified aminolipid, distinguishing it from *A. caldus* KU^T^ or other species of *Acidithiobacillus*. *A. caldus* KU^T^ contains diphosphatidylglycerol, phosphatidylglycerol, phosphatidylethanolamine, an unidentified aminolipid, an unidentified glycolipid and an unidentified lipid (Fig. S1). The major cellular fatty acid of S30A2^T^ is C_19 : 1_ω7*c* (42.9 %) which is similar to the reference strain *A. caldus* KU^T^ with C_19 : 1_ω7*c* (43.5 %), cyclo-C_19 : 0_ω8c (11.4 %) and C_18 : 1_2-OH (10.6 %), but the contents of fatty acids component are different. Detailed fatty acid profiles of strain S30A2^T^ and other type strains of *Acidithiobacillus* are shown in Table 1. Previous studies have indicated that UQ-8 is the main respiratory quinone of all members of the genus *Acidithiobacillus* [1]. In our study, UQ-8 (67.7 %) and UQ-7 (32.3 %) were detected as the main respiratory quinones of S30A2^T^, rather than, UQ-8 (93.5 %), UQ-7 (6.2 %) and UQ-9 (0.3 %), which were detected in *A. caldus* KU^T^.

In conclusion, strain S30A2^T^ is considered to be a novel species within the genus *Acidithiobacillus* based on the results of the phylogenetic analysis, genomic data, as well as physiological and chemotaxonomic features, for which the name *Acidithiobacillus acidisediminis* sp. nov. is proposed.

## Description of *Acidithiobacillus acidisediminis* sp. nov.

*Acidithiobacillus acidisediminis* (a. ci. di. se. di’ mi. nis. L. masc. adj. *acidus*, sour; L. neut. *sedimen*, sediment; N.L. gen. n. *acidisediminis*, of an acidic sediment).

Cells are Gram-stain-negative, non-endospore forming, highly motile, rod-shaped (0.3–0.9×0.7–1.3 µm), and both flagella and pili are observed by transmission electron microscopy. Colonies grown on solid isolation medium at 38 °C for 7 days are milky white, opaque, smooth, circular swollen and easy to pick up. Strain S30A2^T^ is acidophilic, with growth at pH 2.0–4.5 (optimum, pH 2.5), and mesophilic, with growth at 30–50°C (optimum, 38 °C). Cells can grow in medium with NaCl concentrations ranging from 0 to 4 % (w/v). Oxidase and catalase activity are negative. The strain grows obligately aerobically; precipitated sulphur and tetrathionate can be used as electron donors, but thiosulphate or sulphite cannot. The type strain failed to oxidize or reduce iron or metal sulphide. The polar lipids of S30A2^T^ contain two unidentified phospholipids, three unidentified aminophospholipids and an unidentified aminolipid. The predominant cellular fatty acid of S30A2^T^ is C_19 : 1_ω7*c* and the main respiratory quinones are UQ-8 and UQ-7. The genomic DNA G+C content of the strain is 59.25 mol%. The average nucleotide identity ANIb and ANIm values between strain S30A2^T^ and *A. caldus* KU^T^ are 70.95 and 89.78 %, respectively and the dDDH value is 24.9 %.

The type strain, S30A2^T^ (=CGMCC 1.17059^T^=KCTC 72580^T^), was isolated from sediment of acid mine drainage environment of a copper mine in Mengzi City, Yunnan Province, PR China.

## supplementary material

10.1099/ijsem.0.005868 Uncited Fig. S1.

10.1099/ijsem.0.005868 Uncited Supplementary Material 1.

## Abbreviations

AGL: aminoglycolipid
AL: aminolipid
ANIb: average nucleotide identity using BLAST
ANIm: average nucleotide identity using MUMmer
APL: aminophospholipid
CRISPR: Clustered Regularly Interspaced Short Palindromic Repeat Sequences
dDDH: digital DNA-DNA hybridization
DPG: diphosphatidylglycerol
GIs: genomics islands
GL: glycolipid
ML: maximum-likelihood
PE: phosphatidylethanolamine
PG: phosphatidylglycerol
PL: phospholipid
PME: phosphatidylmethylethanolamine
UQ: ubiquinone
