# Supplementary figures and images for: Acidithiobacillus acidisediminis sp. nov., an acidophilic sulphur-oxidizing chemolithotroph isolated from acid mine drainage sediment
Source: Int J Syst Evol Microbiol. 2024 May 28;74(5):005868. doi: 10.1099/ijsem.0.005868 (PMC11165880; doi:10.1099/ijsem.0.005868)

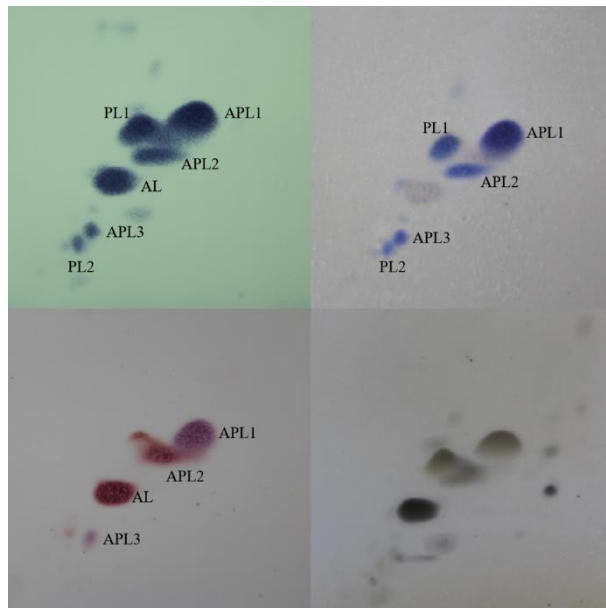

(a)

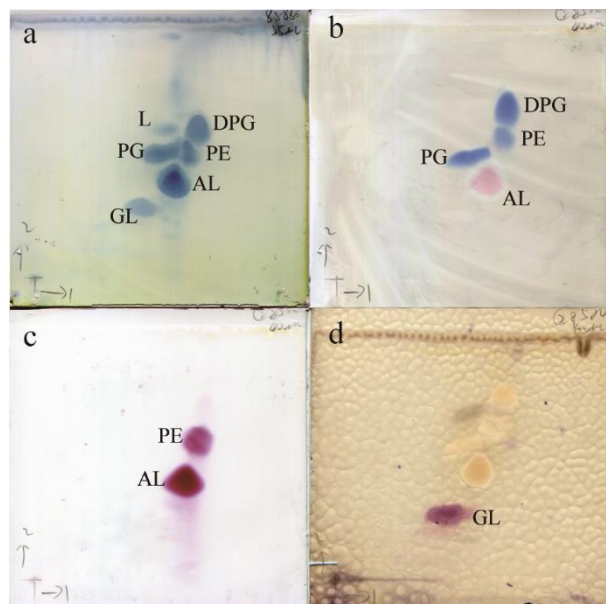

(b)

Supplement: Uncited Fig. S1. [file ijsem-74-05868-s001.pdf]
