# Supplementary material for: Acidithiobacillus acidisediminis sp. nov., an acidophilic sulphur-oxidizing chemolithotroph isolated from acid mine drainage sediment
Source: Int J Syst Evol Microbiol. 2024 May 28;74(5):005868. doi: 10.1099/ijsem.0.005868 (PMC11165880; doi:10.1099/ijsem.0.005868)
Supplement: Uncited Supplementary Material 1. [file ijsem-74-05868-s002.pdf]

International Journal of Systematic and Evolutionary Microbiology (IJSEM)

Supplementary Material

Acidithiobacillus acidisediminis sp. nov., an acidophilic sulfur-oxidizing chemolithotroph isolated from sediment of acid mine drainage

Xiu-Tong Li[1,2], Zong-Lin Liang[1,2], Ye Huang[1,2], Zhen Jiang [1,2], Zhen-Ni Yang[1,2], Nan Zhou[1], Ying Liu[1], Shuang-Jiang Liu[1,2,3,4*], Cheng-Ying Jiang[1,2,3*]

[1] State Key Laboratory of Microbial Resources, Institute of Microbiology, Chinese Academy of Sciences, Beijing 100101, China

[2] University of Chinese Academy of Sciences, Beijing 100049, China

[3] Innovation Academy for Green Manufacture, Chinese Academy of Sciences, Beijing 100190, China

[4] State Key Laboratory of Microbial Biotechnology, Shandong University, Tsingdao 266237, China

[*]Corresponding authors:

Prof. Cheng-Ying Jiang; E-mail: jiangcy@im.ac.cn

Prof. Shuang-Jiang Liu; E-mail: Liusj@im.ac.cn

Institute of Microbiology, Chinese Academy of Sciences, No.1, Beichen West Road, Chaoyang District, Beijing 100101, China

Tel: +86-10-64807423; Fax: +86-10-64807421

Subject Category: New taxa (*Pseudomonadota*)

Running title: *Acidithiobacillus acidisediminis* sp. nov.

Keywords: *Acidithiobacillus acidisediminis*; sulfur-oxidizing; sediment of acid mine drainage; acidophile

Figure S1. Polar lipid profile of strain S30A2^{T} (a) and *Acidithiobacillus caldus* KU^{T} (b) by two-dimensional thin-layer method. Annotation: a. total polar lipids; b. phospholipids; c. aminolipids; d. glycolipids. Abbreviations: PL1–2, unidentified phospholipids; GL, unidentified aminolipid; APL1–3, unidentified aminophospholipids; DPG, diphosphatidylglycerol; PG, phosphatidylglycerol; PE, phosphatidylethanolamine; AL, unidentified aminolipid; L, unidentified lipid.

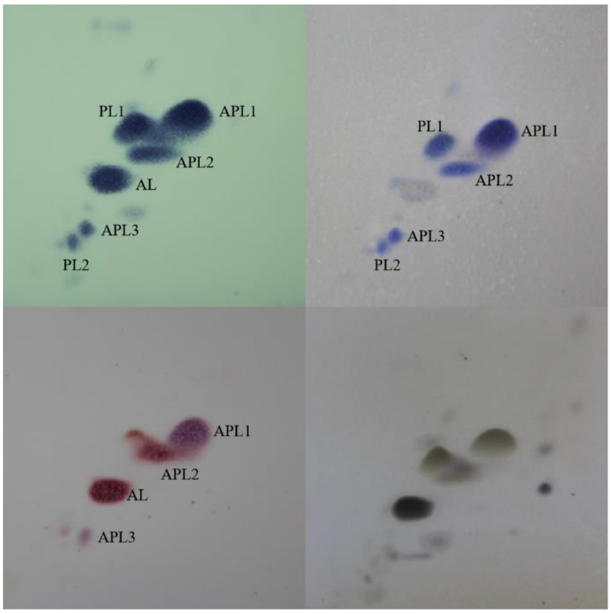

(a)

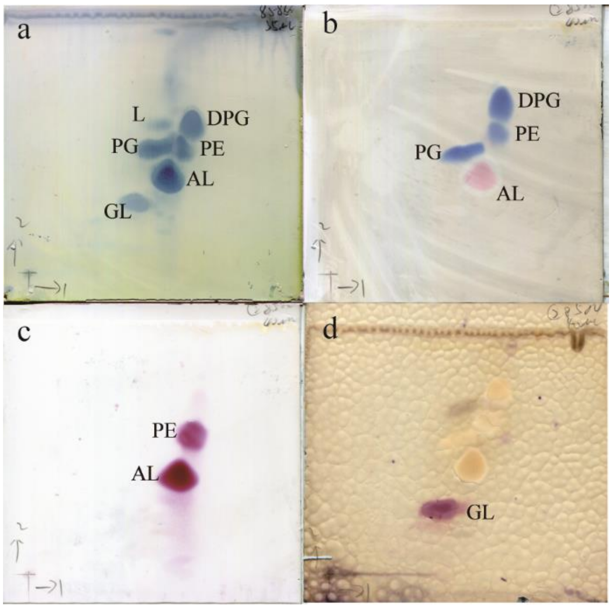

(b)

Table S1. The COGs functional categories of the protein-coding genes of strain $S30A2^{T}$.

**Function code**	**Number**	**Function**
M	213	Cell wall/membrane/envelope biogenesis
J	186	Translation, ribosomal structure and biogenesis
R	179	General function prediction only
C	177	Energy production and conversion
E	156	Amino acid transport and metabolism
T	151	Signal transduction mechanisms
P	147	Inorganic ion transport and metabolism
L	146	Replication, recombination and repair
S	135	Function unknown
X	121	Mobilome: prophages, transposons
K	118	Transcription
G	116	Carbohydrate transport and metabolism
H	114	Coenzyme transport and metabolism
O	112	Posttranslational modification, protein turnover, chaperones
V	107	Defense mechanisms
N	92	Cell motility
I	72	Lipid transport and metabolism
F	70	Nucleotide transport and metabolism
U	70	Intracellular trafficking, secretion, and vesicular transport
D	44	Cell cycle control, cell division, chromosome partitioning
Q	25	Secondary metabolites biosynthesis, transport and catabolism
W	25	Extracellular structures
A	1	RNA processing and modification
B	1	Chromatin structure and dynamics
Y	0	Nuclear structure
Z	0	Cytoskeleton

Table S2. Antibiotic sensitivities of strain S30A2^{T} and *Acidithiobacillus caldus* KU^{T}.

Symbols: +, sensitive; −, insensitive; W, weakly sensitive. Data are from this study.

**Antibiotic**	**Content**	**S30A2^{T}**	**KU^{T}**	**Antibiotic**	**Content**	**S30A2^{T}**	**KU^{T}**
Amoxicillin	10 μg	+	W	Ceftizoxime	30 μg	+	–
Ampicillin	10 μg	+	–	Cefuroxime	30 μg	W	–
Azithromycin	15 μg	–	–	Cephalothin	30 μg	W	–
Azlocillin	75 μg	+	W	Chloramphenicol	30 μg	–	–
Carbenicillin	100 μg	+	W	Clindamycin	2 μg	–	–
Cefaclor	30 μg	+	+	Gentamicin	10 μg	–	–
Cefazolin	30 μg	–	–	Kanamycin	30 μg	–	–
Cefepime	30 μg	+	–	Mezlocillin	75 μg	+	W
Cefetamet	30 μg	W	–	Neomycin	30 μg	–	–
Cefixime	5 μg	W	–	Oxacillin	1 μg	–	–
Cefmetazole	30 μg	–	–	Piperacillin	100 μg	+	W
Cefodizime	30 μg	+	–	Polymyxin B	300 IU	–	–
Cefoperazone	75 μg	W	–	Rifampin	5 μg	+	–
Cefoperazone	30 μg	–	–	Spectinomycin	100 μg	+	–
Cefotaxime	30 μg	W	–	Vancomycin	30 μg	–	–
Cefpiramide	75 μg	+	–	Netilmicin	30 μg	W	–
Ceftazideme	30 μg	–	–	Aztreonam	30 μg	–	–
